# Contribution of fibrin glue in the surgery of cyanogenic and non-cyanogenic congenital cardiopathies: retrospective cohort study

**DOI:** 10.1186/s12872-019-1102-7

**Published:** 2019-05-16

**Authors:** Amine Cheikh, Mohamed Rida Ajaja, Hicham Rhazali, Mustapha Bouatia, Ali Benomar, Anas Slaoui, Yahia Cherrah, Redouane Abouqal, Amine El Hassani, Younes Cheikhaoui

**Affiliations:** 1 Abulcasis University, Faculty of Pharmacy, Department of Pharmacy, Cheikh Zaid Hospital, Rabat, Morocco; 2 Department of Pediatric Cardiac Surgery, Cheikh Zaid Hospital, Rabat, Morocco; 3 Department of Intensive Care, Cheikh Zaid Hospital, Rabat, Morocco; 4 Mohammed V University, Faculty of Medicine and Pharmacy, Rabat, Morocco; 5 Department of Neurology, Abulcasis University, Faculty of Medicine, Rabat, Morocco; 6 Department of Cardiac Surgery, Abulcasis University, Faculty of Medicine, Rabat, Morocco; 7 Laboratory of Epidemiology and Clinical Research, Mohammed V University, Rabat, Morocco; 8 Mohammed V University, Faculty of Medicine and Pharmacy, Cheikh Zaid Hospital, Rabat, Morocco

**Keywords:** Fibrin glue, Cyanogenic, Non-cyanogenic, Congenital cardiopathies

## Abstract

**Background:**

Postoperative bleeding in cardiovascular surgery is a frequent and complicated situation for the surgical team, and may also be responsible for significant hospital expenditures. Fibrin glue are indicated in surgery to improve hemostasis when conventional techniques such as compression, sutures or electrocoagulation are insufficient. Through this study, we tried to study the contribution of fibrin glue to the improvement of the clinical parameters (volume of postoperative bleeding, length of stay in intensive care, volume of blood transfusion ...) in two populations having undergone cardiac surgery, one in which we used the fibrin glue and one without fibrin glue.

**Methods:**

This was a retrospective cohort study conducted in the cardiovascular surgery department of our Hospital in Rabat between June 2012 and June 2015. Fibrin glue (Tissucol® of BAXTER) was used in one group with an haemostatic aim. The pre and post-operative clinical data of the patients were analyzed and compared with data from patients who were operated without the use of fibrin glue because it was not yet available in the hospital. The clinical parameters were collected analyzed using the SPSS 13.0 software.

**Results:**

One hundred ten patients were included in this study. The fibrin glue was used intraoperatively in 55 patients and not used in 55 patients. 43 (39.1%) had cyanogenic diseases and 67 (60.9%) had non-cyanogenic pathologies. The volume of transfused red blood cells was lower in patients in whom we used biological glue (*p* = 0.005), as well as the number of days spent in intensive care (*p* = 0.02). However, the difference was not significant between the two groups for other parameters such as bleeding volume per kg, the number of units of fresh frozen plasma and the platelet units count transfused.

**Conclusions:**

The results we found show that fibrin glue reduces the duration of hospitalization in resuscitation and reduces the number of units of transfused red blood cells to patients after surgery. However, it does not reduce significantly the total postoperative bleeding volume per weight, the number of fresh frozen plasma units or platelets units transfused. The fibrin glue could therefore be of moderate benefit in pediatric cardiac surgery.

## Background

Cardiovascular surgery in children is a surgical procedure with a high risk of haemorrhage. This is due to several parameters: 1) cardiothomy: approach to large vessels (aorta and vena cava), 2) the technique used: Extracorporeal circulation induces hemodilution, depletion of coagulation factors, platelet dysfunction. The consequence of these elements is the occurrence of significant postoperative bleeding and pathophysiological abnormalities 3) the patient’s low age; and 4) the low blood volume of the patients, ie any bleeding could represent an emergency situation for the surgical team.

Hemostasis is an important physiological procedure for healing wounds and minimizing bleeding, this procedure achieved through the successive stages of physiological coagulation. Rapid and efficient haemostasis is very important for most patients as well as for the medical profession and the health system in general, especially to health insurance funds that reimburse patients. In surgical practice, bleeding control pre- and intra-operative reduces significant blood loss and thus helps to minimize postoperative complications of bleeding (eg, transfusion with associated risk) [1], postoperative infection [2], prolonged hospital stay times).

The use of some fibrin-based biological glues is associated with significant improvement in patients with some heart problems [3]. Biological glue has been widely used in Europe in several surgical procedures, including cardiovascular surgery [4]. Its use has been associated with several advantages such as reduced operating time, minimization of localized bleeding, reduction of postoperative blood loss and reduction of the incidence of surgical revision following bleeding [5]. Fibrin glue is used in cardiovascular surgery for several reasons: tissue sealing to control difficult bleeding on vascular anastomoses, promote hemostasis in patients under heparin, the use of highly porous vascular grafts, control of residual bleeding after embolization and surgical resection and during surgical operations to treat congenital heart defects [6] it also facilitates thrombosis of an expanding abdominal aortic aneurysm [7].

Conventional methods are generally used to properly control bleeding that occurs during cardiovascular surgeries. Unfortunately, these means are often associated with prolonged duration of intervention and persistent hemorrhages. They may also be associated with cardiovascular instability that may increase perioperative morbidity and mortality [8].

The use of biological sealants has been promoted primarily based on their ability to enhance coagulation in addition to their capacity to create a mechanical barrier at the site of bleeding [9].

The objective of our work is to study the contribution of fibrin glue to the improvement of the clinical parameters in the surgery of cyanogenic and non-cyanogenic congenital cardiopathies, especially volume of postoperative bleeding, length of stay in intensive care, volume of blood derivatives transfusion in two populations having undergone cardiac surgery, one in which we used the fibrin glue and one without fibrin glue.

## Methods

This was a retrospective cohort study conducted between January 2012 and December 2015 in the Pediatric Cardiovascular Surgery Unit at the International University Hospital Cheikh Zaid in Rabat.

We compared two groups of patients operated by the same team to treat congenital heart diseases. The first group was operated for congenital heart disease without the use of fibrin glue and the second group using the fibrin glue after its introduction to the hospital.

The pre- and post-operative clinical data of the patients were analyzed. The hospital information system was used for this purpose. The main parameters and indicators analyzed were: the volume of post-operative bleeding, the use of blood transfusion and its derivatives, and the length of stay in intensive care inpatients.

Continuous variables are expressed as either a mean or median and categorical variables are expressed as a percentage. The comparison was made using the non-parametric tests of Mann Whitney or the Student test. Correlations were also made to compare the quantitative variables. The statistical analysis was done using the SPSS 13.0 software.

## Results

One hundred ten patients were included in this study (20 patients were excluded because we did not have complete data in their medical records, 9 in the group of fibrin glue and 11 in the group without fibrin glue). The fibrin glue was used intraoperatively in 55 patients and not used in 55 patients. 43 (39.1%) had cyanogenic diseases and 67 (60.9%) had non-cyanogenic pathologies. The median age of our population is 405 days [180–1822.5]. Inter ventricular communication, atrioventricular duct and Tetralogy of Fallot, are the most frequent congenital cardiopathies in our series (24.5, 22.7, 21.8% respectively). The demographic and clinical characteristics of the study population are summarized in Table 1.

**Table 1:** Demographic and clinical characteristics of the study population

Criterion	With fibrin glue	Without fibrin glue	p
Sex			0.28
F	22 (40%)	26 (47.3%)	
M	33 (60%)	29 (52.7%)	
Age (median in day)	360 [180–2160]	510 [210–1800]	0.60
Weight (median in kg)	7.7 [5–15]	9.2 [6–15]	0.32
Type of pathology			0.35
cyanogenic	20 (36.4%)	23 (41.8%)	
Non-cyanogenic	35 (63.6%)	32 (58.2%)	
Total bleeding volume (median in mL)	132 [70–200]	118 [80–220]	0.96
bleeding volume/kg (median in mL/kg)	14.1 [10–24.3]	15.6 [7–21]	0.39
Length of stay in intensive care	2 [2–3]	3 [2–4]	0.02
Number of units of transfused red blood cells	2 [2–3]	3 [2–3]	0.005
Number of units of fresh plasma frozen transfusion	3 [2–3]	2.9 [3–3]	0.48
Number of units of platelets transfused	0 [0–2]	0 [0–0]	0.39

The volume of transfused red blood cells units was lower in patients in whom we used fibrin glue (2 vs 3 units, *p* = 0.005), as well as the number of days spent in intensive care (2 vs 3 days, *p* = 0.02). However, the difference was not significant between the two groups for other parameters such as bleeding volume per kg (14.1 vs 16 ml/kg, *p* = 0.39), the number of units of fresh frozen plasma (3 vs 3 units, *p* = 0.48) and the platelet units transfused (1 vs 0.7, *p* = 0.39).

The volume of bleeding increases with age significantly (r = 0.455, *p* < 0.001), with weight (r = 0.369, *p* < 0.001) and with CEC time (r = 0.281, *p* = 0.003).

## Discussion

Although the safety of hemostatic fibrin sealants and their efficacy in reducing blood loss has been demonstrated [10, 11], there is a relative shortage of prospective clinical trials, particularly in pediatric cardiac surgery [12].

For pediatric cardiac surgery, the need for perfect hemostasis is even greater due to the high incidence of coagulopathy following open-heart procedures [13] and increased risks associated with the use of blood and blood products.

Our series is the largest series that has dealt with this subject at the national level and one of the largest at the international level. Fibrin glue was not available on the Moroccan market before 2012. After several requests, BAXTER took charge of the importation of the product and its introduction on the national market.

We tried to study the contribution of fibrin glue “Tissucol®” in patients who underwent cardiovascular surgery for congenital heart disease by comparing some clinical parameters between the two groups. The selected indicators are those found in the literature and are related to the bleeding and indications for fibrin glue. Bleeding by weight, hospital stay in intensive care after surgery, number of transfused units of red blood cells, fresh frozen plasma and platelet, all of these parameters were investigated and compared between both groups.

The results of our study are broadly similar to those in the literature. Thus, we found that the use of fibrin glue allowed the reduction of the number of units of red blood cells transfused to the patients after surgery, it also made it possible to reduce the stay of hospitalization in intensive care after surgery. However, the result that remains contradictory with those found in the literature is the insignificant difference in the volume of bleeding between the two groups. This is probably due to the difference between the two groups regarding demographic characteristics and the type of pathologies and surgery.

Twenty-four trials have been reported by Kjaergard and Fairbrother about the use of fibrin adhesives in cardiac surgery. Four studies reported no difference and 20 trials reported positive results in decreased bleeding [14]. Furthermore, Lamm et al. reported several cases of acute occlusion when the “Tissucol®” fibrin glue was used near the anastomoses. This requires considerable caution when using this product [15]. Other randomized controlled trials compared the use of a variety of topical hemostatic agents with variable efficacy and varying degrees of evidence in favor of their benefits [5, 12, 16–22].

Huth et al. reported that fibrin glue “Tissucol®” was used for local hemostasis in 21 patients undergoing Fallot tetralogy correction (ToF) and in 10 patients undergoing a Senning procedure in transposition of major arteries (TGA). The authors compared postoperative blood loss to 20 ToF patients and 10 TGA patients who had a correction one year ago without fibrin glue and found that two hours after surgery, bleeding with fibrin glue was lower than without fibrin glue (*p* < 0.01) in patients with ToF and TGA. Over the next 18 h, the secretion of thoracic drains was identical in both groups [23]. This result could be considered similar to our result since the overall bleeding volume is similar in both groups. It is likely that fibrin glue only reduces anastomotic bleeding, and after the first couple of hours most of the ongoing blood loss is related to the sternum and to the large raw surface in the mediastinum, which is identical in both groups.

Cyanogenic pathologies, mainly transposition of large vessels, Fallot’s tetralogy, obstacles to the outflow pathway and pulmonary atresia are pathologies that cause more bleeding due to the pathophysiological mechanism of these pathologies and the large number of stitches and arterial remodeling performed. In almost all cases, cardiovascular surgeons use biological glue after sutures and anastomoses. In addition to the hemostatic effect reported in all the above studies, this may have a reassuring effect for the surgical and anesthetist team.

In the case of non-cyanogenic pathologies, for example inter-ventricular communication (IVC), inter-auricular communication (IAC), the atrio-auricular canal (CAV), the risk of bleeding is less than that of cyanogenic pathologies and the use of the biological glue is subject to the appreciation of the surgical team.

Codispoti et al. have showed, in a randomized controlled trial in pediatric cardiac surgery with coagulopathy, that there was a significant reduction in the use of blood products and time spent in the operating room to achieve hemostasis when the fibrin glue was used [12].

We can thus deduce from the above that fibrin glues are mainly used in cardiovascular and cardiothoracic surgery as an adjunct to haemostasis, their effectiveness is reported and confirmed by several teams [24]. Fibrin adhesives are also used in cardiovascular surgery to seal sutures, baselines, anastomoses, fistulas and injured surfaces [25].

This study has three limitations. The first is that it is a retrospective study with all of the risks and bias associated with that type of studies. The second concerns the demographic characteristics (age and weight) of the two populations even if the difference is not significant (*p* = 0.60, *p* = 0.32 respectively), and the third concerns the distribution of the pathologies (cyanogenic and non-cyanogenic) in each group of patients even if the difference is not significant (*p* = 0.35).

## Conclusions

In pediatric cardiac surgery, bleeding represents a dangerous situation that can complicate the surgical procedure and the post-operative follow-up especially in cyanogenic pathologies and pathologies requiring a significant number of sutures and remodeling.

Fibrin sealants can reduce postoperative complications by promoting hemostasis and the avoidance of blood loss. The reduction in hemorrhage also reduces the need for blood transfusions, which may lead to a reduced risk of viral infection.

## Abbreviations

CAV: Atrio-auricular canal
IAC: Inter-auricular communication
IVC: Inter-ventricular communication
TGA: Transposition of major arteries
ToF: Fallot tetralogy correction

## References

[1] Tong M, El-Farra NS, Reikes AR, Co RL (1995). Clinical outcomes after transfusion-associated hepatitis C. N Engl J Med.

[2] Murphy PJ, Connery C, Hicks GL, Blumberg N (1992). Homologous blood transfusion as a risk factor for postoperative infection after coronary artery bypass graft operations. J Thorac Cardiovasc Surg.

[3] Rousou JA (2013). Use of fibrin sealants in cardiovascular surgery: a systematic review. J Card Surg.

[4] Borst H, Haverich A, editors. Fibrin seal in cardiovascular surgery: proceedings of an international workshop, Hannover, Germany, Nov 14, 1981. Thorac Cardiovasc Surg. 1982;30:195–241.

[5] Rousou J, Levitsky S, Gonzalez-Lavin L, Cosgrove D, Magilligan D, Weldon C, Hiebert C, Hess P, Joyce L, Bergsland J (1989). Randomized clinical trial of fibrin sealant in patients undergoing resternotomy or reoperation after cardiac operations. A multicenter study. J Thorac Cardiovasc Surg.

[6] Stark J, de Leval M (1984). Experience with fibrin seal (Tisseel) in operations for congenital heart defects. Ann Thorac Surg.

[7] Vercellio G, Coletti M, Agrifoglio G, Schlag G, Red H (1986). Experience with fibrin glue (Tissucol/Tisseel) in vascular surgery. Fibrin Sealant in OperativeMedicine, Thoracic Surgery- Cardiovascular Surgery.

[8] Basu S, Marini CP, Bauman FG, Shirazian D, Damiani P, Robertazzi R, Jacobowitz IJ, Acinapura A, Cunningham JN (1995). Comparative study of biological glues: cryoprecipitate glue, two-component fibrin sealant, and “French” glue. Ann Thorac Surg.

[9] Guilmet G, Laurian C, Gigou F (1977). La colle gelatine-resorcine-formaldehyde en chirurgie vasculaire. Nouv Press Med.

[10] Sierra DH (1993). Fibrin sealant adhesive systems: a review of their chemistry, material properties and clinical applications. J Biomater Appl.

[11] Mankad PS, Codispoti M (2001). The role of fibrin sealants in hemostasis. Am J Surg.

[12] Mankad PS, Codispoti M (2002). Significant merits of a fibrin sealant in the presence of coagulopathy following paediatric cardiac surgery: randomised controlled trial. Eur J Cardiothorac Surg.

[13] Chan AK, Leaker M, Burrows FA, Williams WG, Gruenwald CE, Whyte L, Adams M, Brooker LA, Adams H, Mitchell L, Andrew M (1997). Coagulation and fibrinolytic profile of paediatric patients undergoing cardiopulmonary bypass. Thromb Haemost.

[14] Kjaergard H, Fairbrother J (1996). Controlled clinical studies of fibrin sealant in cardiothoracic surgery-a review. Eur J Cardiothorac Surg.

[15] Lamm P, Adelhard K, Juchem G (2007). Fibrin glue in coronary artery bypass grafting operations: casting out the devil with Beelzebub?. Eur J Cardiothorac Surg.

[16] Sirlak M, Eryilmaz S, Yazicioglu L (2003). Comparative study of microfibrillar collagen hemostat (Colgel) and oxidized cellulose (Surgicel) in high transfusion-risk cardiac surgery. J Thorac Cardiovasc Surg.

[17] CoStasis Multi-center Collaborative Writing Committee (2001). A novel collagen-based composite offers effective hemostasis for multiple surgical indications: results of a randomized controlled trial. Surgery..

[18] Lumsden AB, Heyman ER (2006). Prospective randomized study evaluating an absorbable cyanoacrylate for use in vascular reconstructions. J Vasc Surg.

[19] Hagberg RC, Safi HJ, Sabik J, Conte J, Block JE (2004). Improved intraoperative management of anastomotic bleeding during aortic reconstruction: results of a randomized controlled trial. Am Surg.

[20] Coselli JS, Bavaria JE, Fehrenbacher J, Stowe CL, Macheers SK, Gundry SR (2003). Prospective randomized study of a proteinbased tissue adhesive used as a hemostatic and structural adjunct in cardiac and vascular anastomotic repair procedures. J Am Coll Surg.

[21] Oz MC, Cosgrove DM, Badduke BR (2000). Controlled clinical trial of a novel hemostatic agent in cardiac surgery. The Fusion Matrix Study Group. Ann Thorac Surg.

[22] Weaver FA, Lew W, Granke K, Yonehiro L, Delange B, Alexander WA (2008). A comparison of recombinant thrombin to bovine thrombin as a hemostatic ancillary in patients undergoing peripheral arterial bypass and arteriovenous graft procedures. J Vasc Surg.

[23] Huth C, Seybold-Epting W, Hoffmeister HE (1983). Local hemostasis with fibrin glue after intracardiac repair of tetralogy of Fallot and transposition of the great arteries. Thorac Cardiovasc Surg.

[24] Von Oppell UO, Zilla P (1998). Tissue adhesives in cardiovascular surgery. J Long Term Effects Med Implants.

[25] Brennan M (1991). Fibrin glue. Blood Rev.

